# Supplementary material for: Limited influence of irrigation on pre-monsoon heat stress in the Indo-Gangetic Plain
Source: Nat Commun. 2022 Jul 25;13:4275. doi: 10.1038/s41467-022-31962-5 (PMC9314405; doi:10.1038/s41467-022-31962-5)
Supplement: Supplementary file 1 — Supplementary Information [file 41467_2022_31962_MOESM1_ESM.pdf]

Limited influence of irrigation on pre-monsoon heat stress in the Indo-Gangetic Plain

Roshan Jha[1], Arpita Mondal[1,2*], Anjana Devanand[3,4], M. K. Roxy[5], Subimal Ghosh[1,2]

[1]IDP in Climate Studies, Indian Institute of Technology Bombay, Mumbai, 400076, India,

[2]Department of Civil Engineering, Indian Institute of Technology Bombay, Mumbai, 400076, India,

[3]Australian Research Council Centre of Excellence for Climate Extremes, University of New South Wales, Sydney, NSW, Australia,

[4]Climate Change Research Centre, University of New South Wales, Sydney, NSW, Australia,

[5]Centre for Climate Change Research, Indian Institute of Tropical Meteorology, Pune, 411008, India

*Corresponding Author: marpita@civil.iitb.ac.in

Supplementary Information

This supplementary information contains supplementary notes describing the irrigation data preparation for AGR experiment, details about HNG experiment based on Huang et al. irrigation data and parameteriation sensitivity experiment. The supplementary note is followed by supplementary figures and tables.

Supplementary Notes

Supplementary Note 1: Preparation of irrigation datasets for AGR experiment

The WRF-CLM4 model with irrigation, groundwater pumping, and paddy field module requires the following irrigation data inputs: irrigated area fractions of the grid cells, irrigation water use over the region, and the proportion of irrigation water used from surface water or groundwater sources.

The district-wise annual agricultural census data provided by the Directorate of Economics & Statistics, Ministry Of Agriculture and Farmers Welfare, Government of India[1] (https://aps.dac.gov.in/LUS/Public/Reports.aspx) is used to estimate pre-monsoon irrigation data from 2004 to 2016. The annual data includes variables like land-use area, area under the crop, crop irrigated area, and source wise irrigated area for different crops at seasonal/annual scale. The five crops (Rice, Maize, Gram, Sunflower, and Sugarcane) are chosen based on crop production statistics (https://aps.dac.gov.in/APY/Public_Report1.aspx) of pre-monsoon season and crop calendar published in the annual Indian Agricultural Statistics report (Supplementary Table 1).

Here, area under the crop and crop irrigated area for Indo-Gangetic Plain states (Bihar, Uttar Pradesh, Haryana, Punjab, and Rajasthan) are used to estimate the irrigation area fractions of grid cells for five crops (Rice, Maize, Gram, Sunflower and Sugarcane). The source wise irrigated area (groundwater or surface water) is used to prescribe the proportion of groundwater or surface irrigation water use.

In India, the flood irrigation system is widely practiced using surface water or groundwater sources[2]. The seasonal water use (mm/hectare) for flood irrigation of these crops based on field surveys[2] as shown in Supplementary Table 1 are used to calculate the pre-monsoon season irrigation water use (mm/day) for the five states. The data for paddy and non-paddy (all crops except rice/paddy) crops are used to model the three new plant functional types (PFTs) to represent irrigated crops, irrigated paddy fields, and rain-fed paddy fields present in the irrigation module. The rain-fed paddy field data is assumed zero as farmers are mostly dependent on irrigation water during the pre-monsoon season.

Further, the district-level census estimates (irrigation area fraction, irrigation water use and proportion of groundwater and surface irrigation wateruse) are interpolated to a regular grid of spatial resolution 0.5°x0.5° for the model input.

For the rest of the Indian states, agricultural census estimates during pre-monsoon season are not available. Hence, the irrigation area fraction of the grid cells and source irrigated area are taken from the Food and Agricultural Organisation (FAO) Global Map of Irrigated Areas (GMIA)[3], and the irrigation water use is taken from average irrigation water withdrawal data developed by Huang et al.[4] from four water models on a 0.5° grid using the GMIA dataset. Here, irrigation water use estimates from Huang et al.[4] are taken for the period 2004 to 2010, and the data for 2010 is used to prescribe the irrigation during 2011 to 2016 over the model domain except for the five states.

Finally, the daily irrigation data inputs from two different sources are merged at 0.5°x0.5° spatial resolution. The irrigation input files for each pre-monsoon season from 2004 to 2016 contain the following variables.

- Non-paddy irrigation use (mm/day)
- Paddy irrigation use (mm/day)
- Fraction of water from the ground (F_grd)
- Fraction of water from surface (F_Surf)
- Non-paddy irrigated area fraction
- Paddy Irrigated area fraction
- Paddy rainfed area fraction (assumed as zero for pre-monsoon)

Supplementary Note 2: Response of near-surface climate variables to Huang et al. monthly irrigation withdrawal dataset

The irrigation water-use is estimated from average irrigation water withdrawal data developed by Huang et al.[4] from four water models on a 0.5° grid using the GMIA dataset. Here, two sets of seasonal pre-monsoon (Feb-May) simulations are performed for 2004, 2008, 2012, and 2016, with two initial months taken as spin-off time at the horizontal spatial resolution of 30km and 30 vertical levels using coupled WRF-CLM model. Two simulations: CTL (irrigation-off) and HNG (irrigation-on with Huang et al. irrigation data), uses initial and lateral boundary conditions from European Centre for Medium-Range Weather Forecast Interim Re-Analysis. The influence of Huang et al. irrigation data on near-surface variables has higher cooling and

moistening effect than the agricultural census-based data (Supplementary Figure 9). One of the reasons behind the over-estimation of irrigation impact may be the estimation of a higher irrigation requirement during dry April-May months in water models used in Huang et al. study, failing to acknowledge the pre-monsoon irrigation practice in Indo-Gangetic Plain. The variable meteorological response in the HNG experiment over Indo-Gangetic Plain is higher because of the very high irrigation area fraction given by GMIA datasets.

Supplementary Note 3: Sensitivity of irrigation influence to different WRF parameterization schemes

To check the sensitivity of irrigation influence to different WRF parameterization schemes, we employed three combination of PBL-Microphysics-Cumulus parameterization schemes along with a newer version of the Rapid Radiative Transfer Model (RRTMG) for longwave and Dudhia scheme for shortwave radiation represents radiation schemes, and a revised MM5 surface layer scheme based on Monin-Obukhov Similarity Theory is used for surface layer scheme.

a. C1: Mellor-Yamada Nakanishi and Niino Level 3-WRF Double-Moment 6-class scheme- Tiedtke scheme (MYNN3-WDM6-Tiedtke)
b. C2: Mellor-Yamada Nakanishi and Niino Level 3-WRF Single-Moment 6-class scheme-KF scheme (MYNN3-WSM6-KF)
c. C3: Mellor-Yamada Nakanishi and Niino Level 3- WRF Single-Moment 6-class scheme- Grell 3D (MYNN3-WSM6-Grell3D)

Three sets of seasonal pre-monsoon (Feb-May) simulations are performed from 2004 to 2016, with two initial months taken as spin-off time at the horizontal spatial resolution of 30km and 30 vertical levels using coupled WRF-CLM model. All three simulations: CTL (irrigation-off), AGR (irrigation-on with agricultural census-based irrigation data), and MOD (irrigation-on with model-estimated irrigation data), uses initial and lateral boundary conditions from European Centre for Medium-Range Weather Forecast Interim Re-Analysis[41].

The sensitivity test of daily mean temperature (Supplementary Fig. 11), daily maximum temperature (Supplementary Fig. 12), and wet-bulb temperature (Supplementary Fig. 13) response to model-estimated irrigation and agricultural census-based irrigation for a different combination of parameterization schemes show results are quite robust. The error bar diagram shows that the agricultural census-based irrigation has a similar influence with three

parameterization combinations with mean irrigation cooling of 0.37°C for daily mean temperature and 0.5°C for daily maximum temperature and mean moistening effect of 0.26°C for wet-bulb temperature (Supplementary Fig. 14). Further, the model-estimated irrigation influence is over-estimated in all the combinations of parameterization schemes.

References

1. Government of India. *Agricultural Statistics At a Glance 2020. Government of India Ministry of Agriculture Department of Agriculture and Cooperation Directorate of Economics and Statistics* (2020).

2. Fishman, R., Devineni, N. & Raman, S. Can improved agricultural water use efficiency save India's groundwater? *Environ. Res. Lett.* **10**, (2015).

3. Siebert, S., Henrich, V., Frenken, K. & Burke, J. Update of the digital global map of irrigation areas to version 5. *Rheinische Friedrich-Wilhelms-Universituy, Bonn, Ger. Food Agric. Organ. United Nations, Rome, Italy* 171 (2013).

4. Huang, Z. *et al.* Reconstruction of global gridded monthly sectoral water withdrawals for 1971-2010 and analysis of their spatiotemporal patterns. *Hydrol. Earth Syst. Sci.* **22**, 2117–2133 (2018).

Supplementary Figures

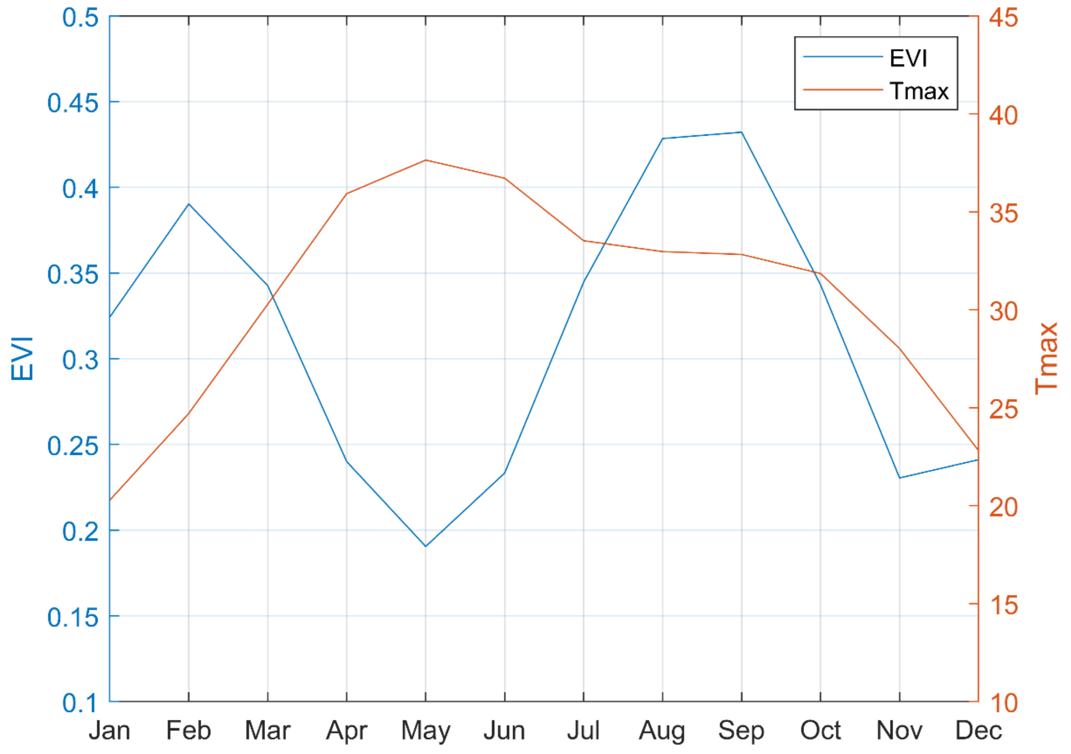

Supplementary Figure 1. Tmax and EVI Climatology. Climatology of Indian Meteorological Department (IMD) observed daily maximum temperature (Tmax) from 1981-2020 and MODIS EVI from 2000-2020 for Indo-Gangetic Plain. Apr–May is pre-monsoon and non-cropping season. Jun–Sep is monsoon and cropping season (Kharif).

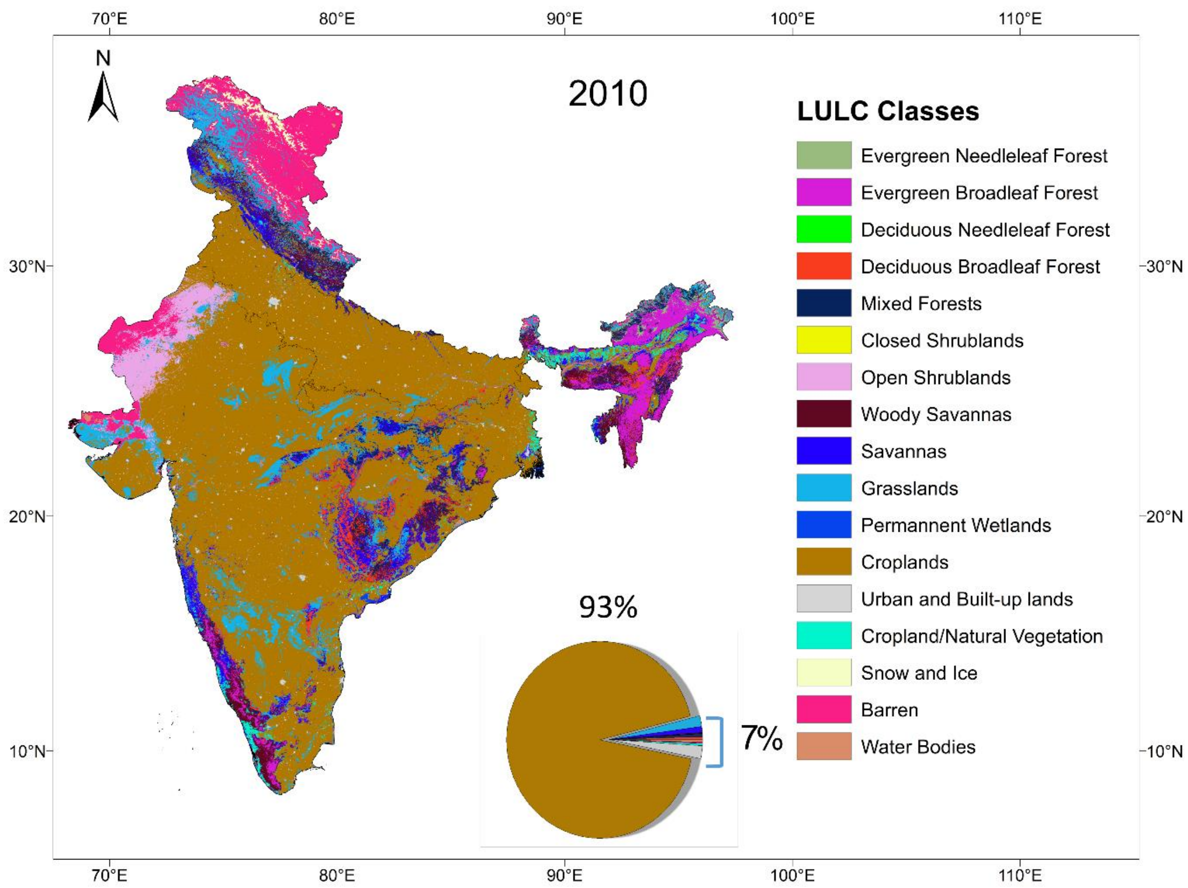

Supplementary Figure 2. Proportion of croplands in Indo-Gangentic Plain. Land use and land cover during 2010 from Moderate Resolution Imaging Spectroradiometer (MODIS) International Geosphere–Biosphere Programme (IGBP) classification scheme at 500m spatial resolution. The pie-chart shows the proportion of different land use land cover in the Indo-Gangetic Plain.

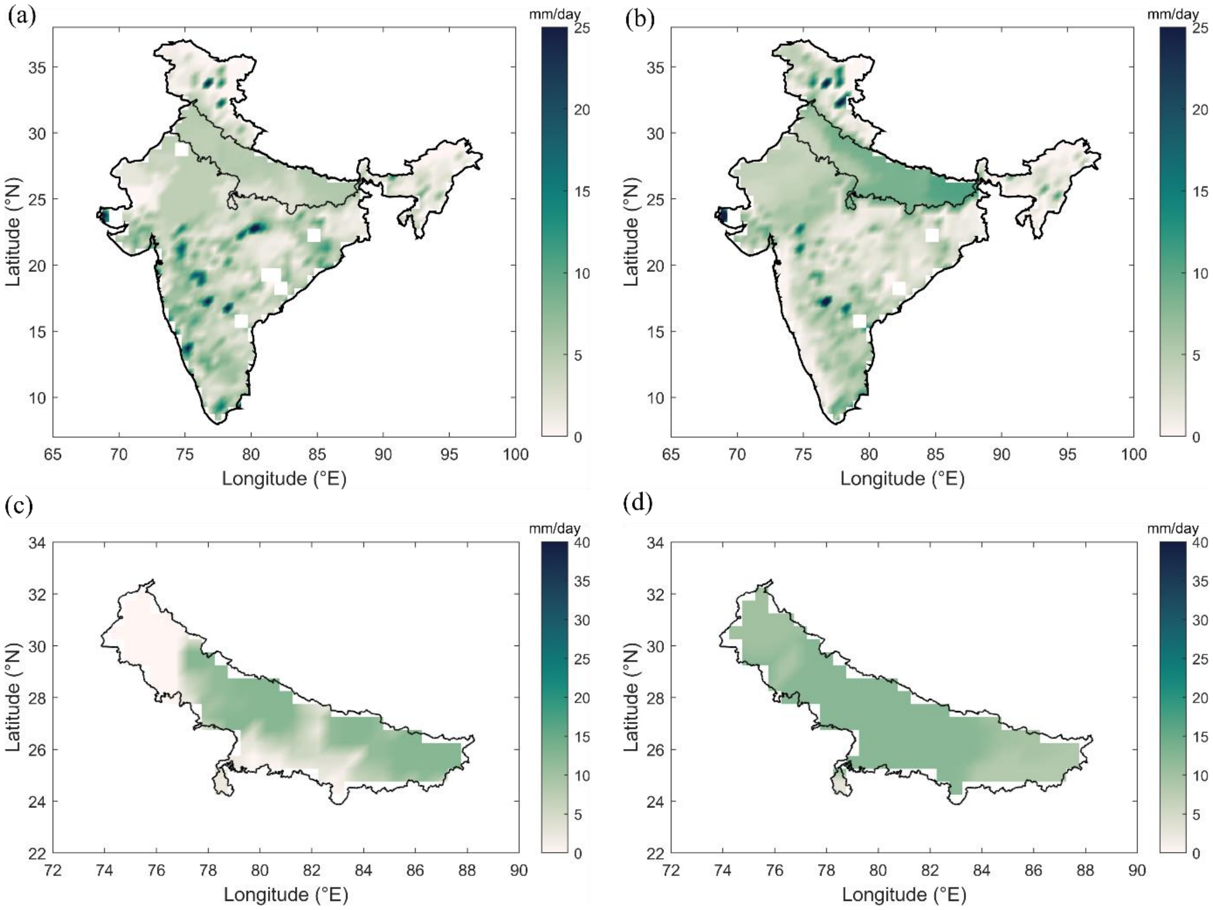

Supplementary Figure 3. Irrigation water use during pre-monsoon and monsoon. Mean (2004-2016) paddy and non-paddy crop irrigation water use from agricultural census-based data. (a) Pre-monsoon non-paddy wateruse (b) Monsoon non-paddy wateruse (c) Pre-monsoon paddy wateruse (d) Monsoon paddy wateruse

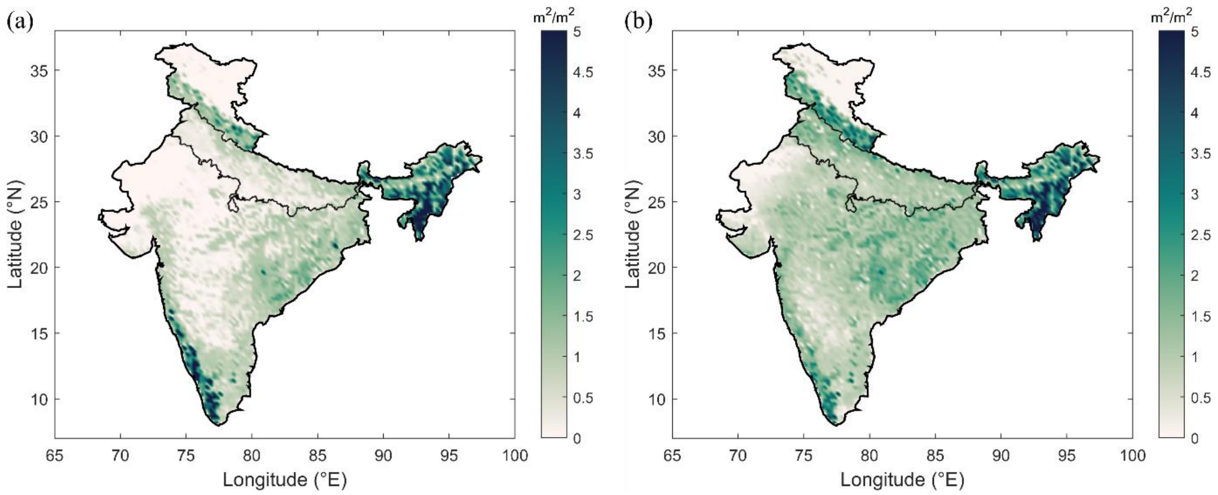

Supplementary Figure 4. Comparison of leaf area index (LAI). Mean LAI (m^2/ m^2) for the period 2001-2020 from Moderate Resolution Imaging Spectroradiometer (MODIS) for (a) Pre-monsoon (b) Monsoon

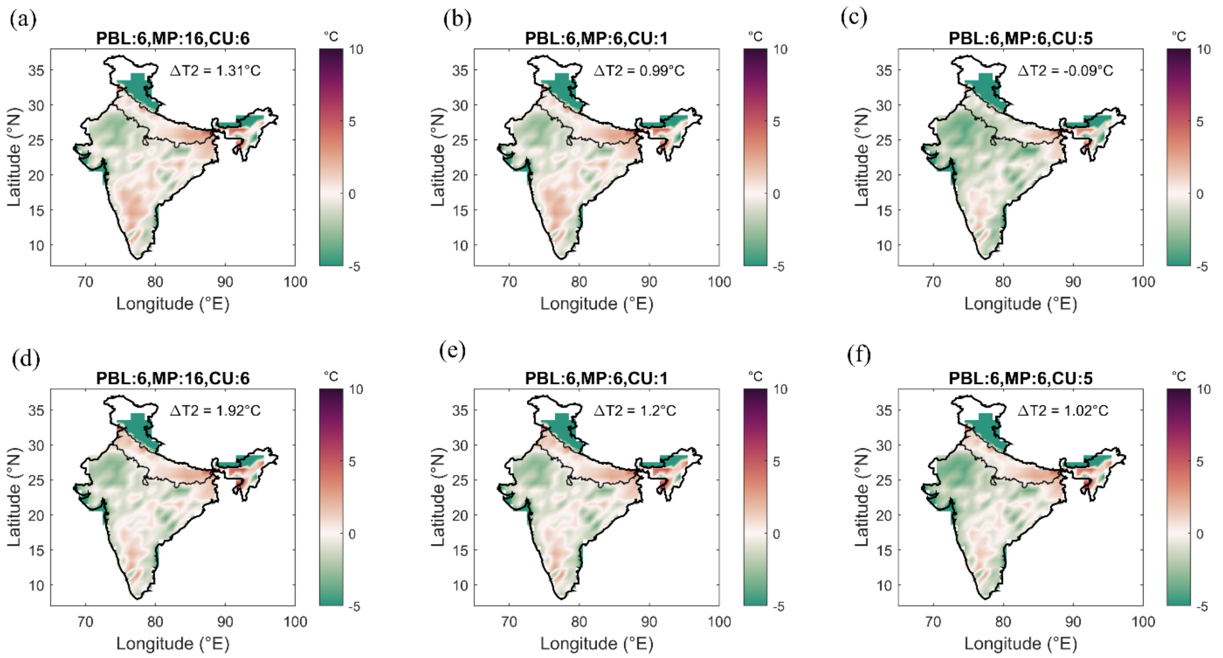

Supplementary Figure 5. Comparison of model simulated daily temperature data with IMD observed data. Difference between AGR simulated and IMD daily mean temperature of April-May for different parameterization combination (C1-PBL:6,MP:16,CU:6; C2-PBL:6,MP:6,CU:1; C3-PBL:6,MP:6,CU:5) for (a,b,c) 2004 (d,e,f) 2006. [C1:MYNN3-WDM6-Tiedtke, C2:MYNN3-WSM6-KF C3:MYNN3-WSM6-Grell3D]

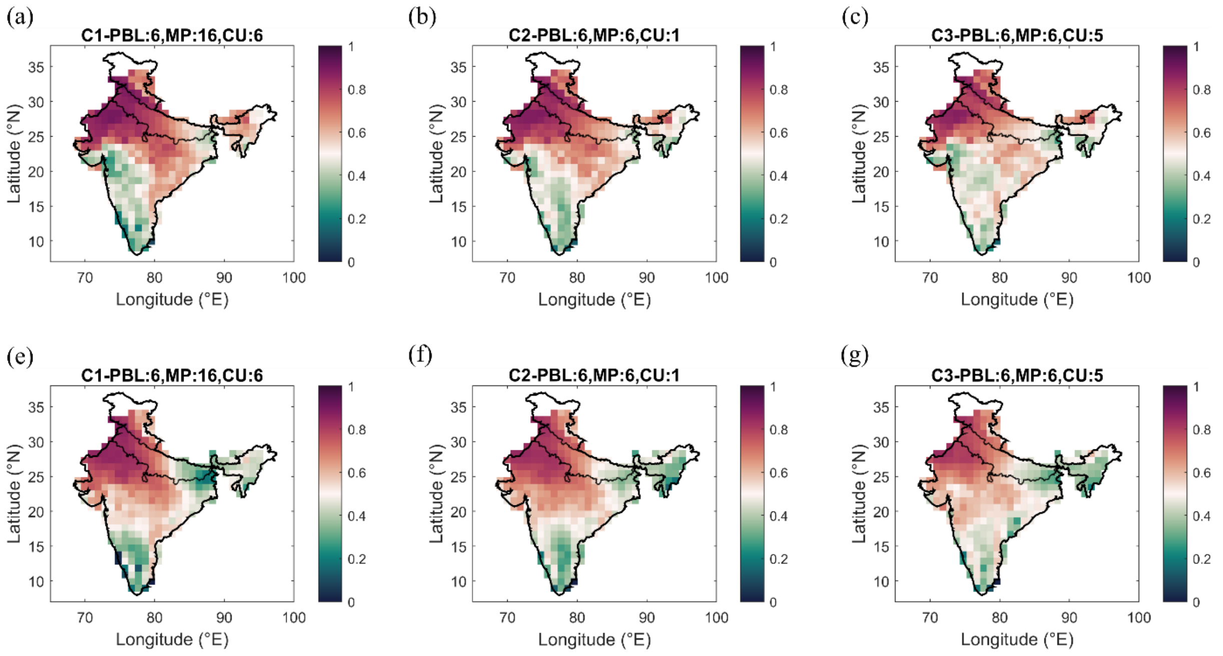

Supplementary Figure 6. Pattern correlation of model simulated daily temperature data with IMD observed data. Correlation coefficient between model simulated with three parameterization scheme (C1, C2 & C3) and IMD observed data for Apr-May (2004-2016) for (a,b,c) T2 and (d,e,f) Tmax. [C1:MYNN3-WDM6-Tiedtke, C2:MYNN3-WSM6-KF C3:MYNN3-WSM6-Grell3D]

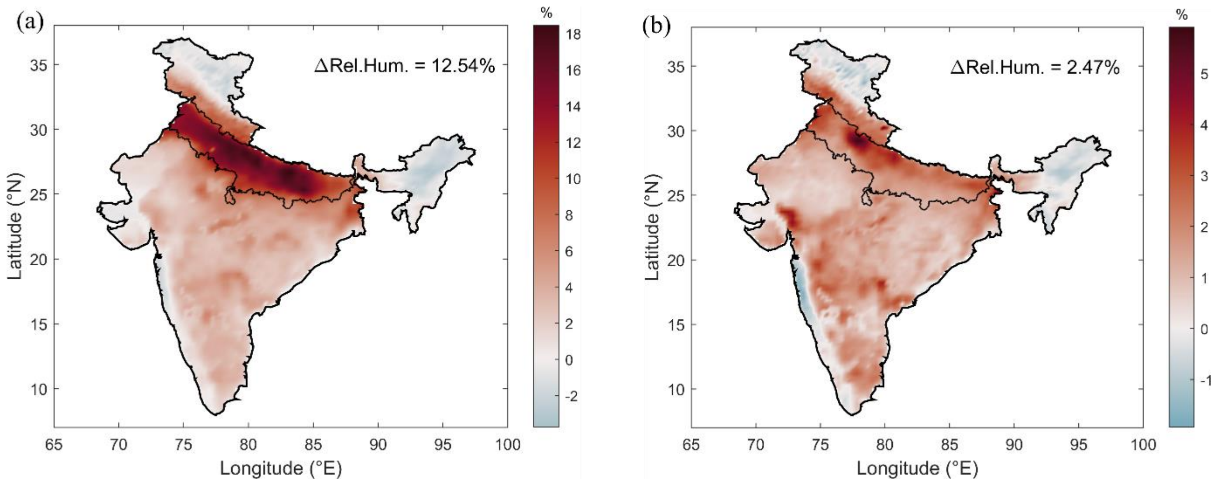

Supplementary Figure 7. Imapct of model-estimated and agricultural census-based irrigation volume on Realtive Humidity. Mean (2004-2016) difference in land surface temperature (°C) between different scenarios (a) Influence of model-estimated Irrigation (MOD - CTL) (b) Influence of agricultural census irrigation (AGR - CTL). The mean difference spatially averaged over Indo-Gangetic Plain is shown as ΔLST. CTL represents WRF-CLM4 simulation with no irrigation, AGR represents WRF-CLM4 simulation with agricultural census-based irrigation data and MOD represents WRF-CLM4 simulation with model-estimated irrigation data.

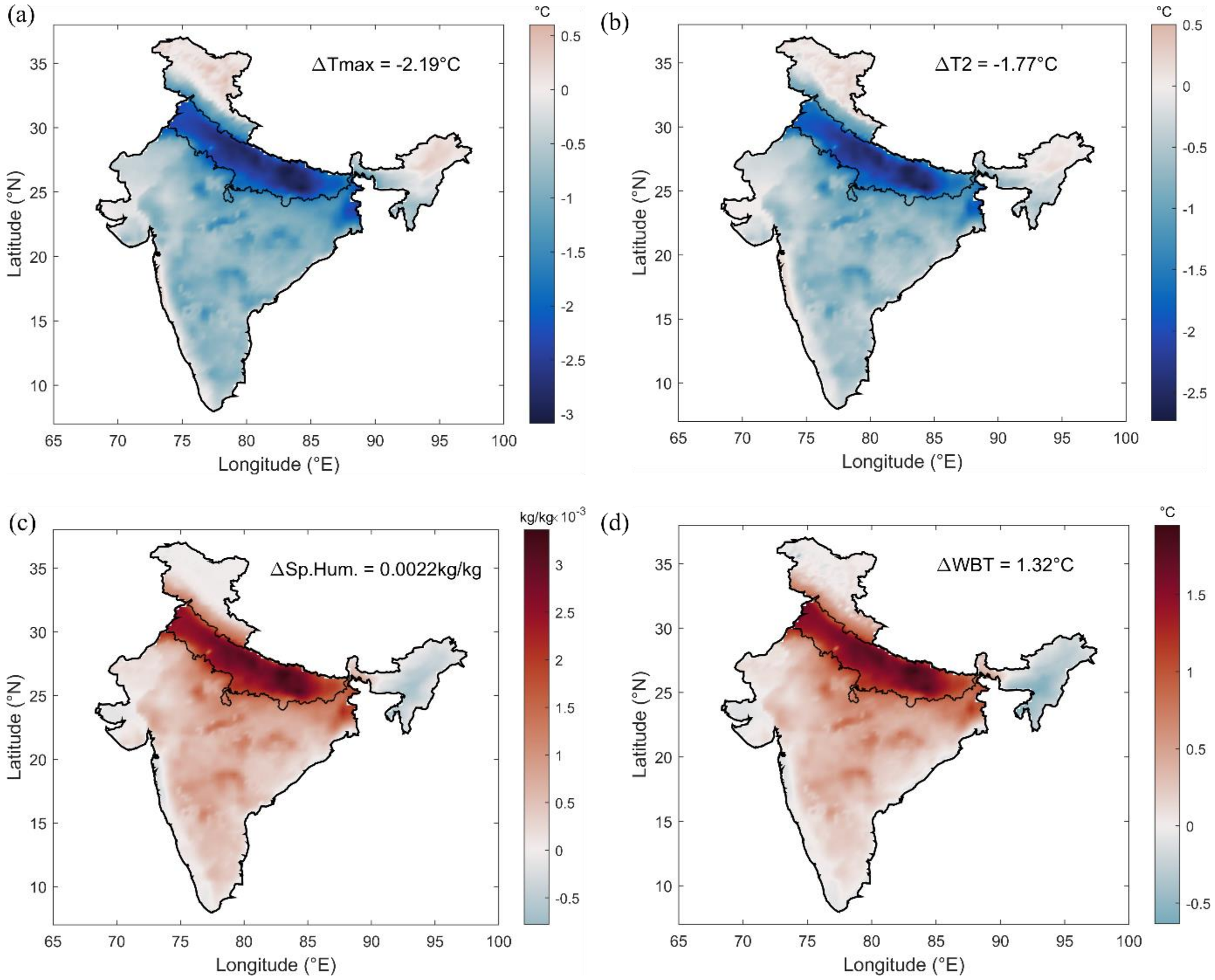

Supplementary Figure 8. Impact of model-estimated irrigation on mean temperature, specific humidity and heat fluxes. Difference between MOD and CTL experiment during pre-monsoon season (April-May) from 2004-2016 for (a) Daily Maximum Temperature (°C) (b) Daily Mean Temperature (°C) (c) Specific Humidity (kg/kg) (d) Wet-bulb Temperature (°C). CTL represents WRF-CLM4 simulation with no irrigation and MOD represents WRF-CLM4 simulation with model-estimated irrigation data.

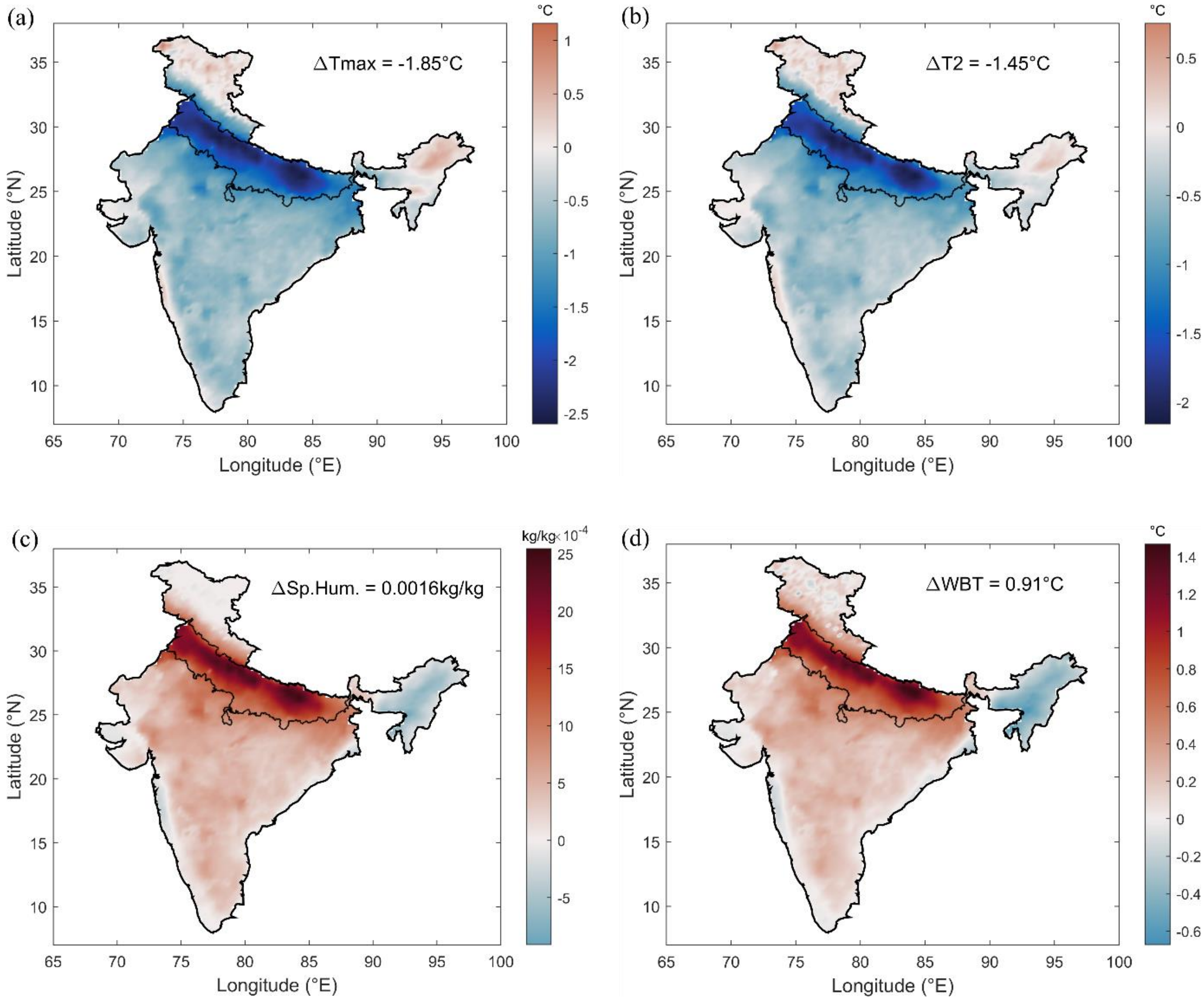

Supplementary Figure 9. Impact of Huang et al. irrigation on daily maximum temperature, daily mean temperature, specific humidity and wet-bulb temperature. Difference between HNG and CTL experiment during pre-monsoon season (April-May) for (a) Daily Maximum Temperature (°C) (b) Daily Mean Temperature (°C) (c) Specific Humidity (kg/kg) (d) Wet-bulb Temperature (°C). CTL represents WRF-CLM4 simulation with no irrigation and HNG represents WRF-CLM4 simulation with Huang et al. monthly irrigation withdrawal data.

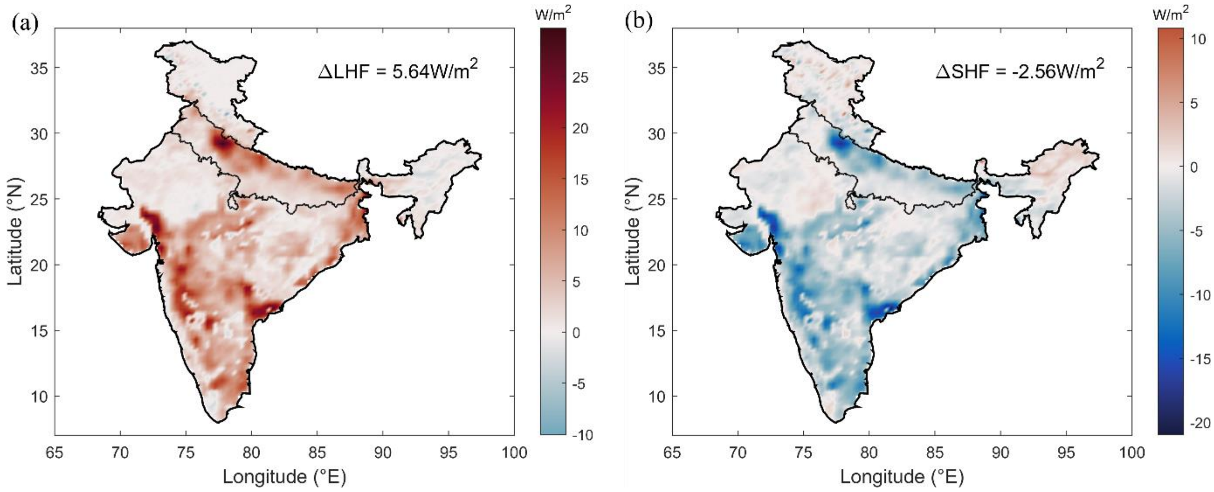

Supplementary Figure 10. Impact of agricultural census based irrigation on heat fluxes. Difference between AGR and CTL experiment during pre-monsoon season (April-May) from 2004-2016 for (a) Latent heat flux (W/m^2) (b) Sensible heat flux (W/m^2). CTL represents WRF-CLM4 simulation with no irrigation and AGR represents WRF-CLM4 simulation with agricultural census-based irrigation data.

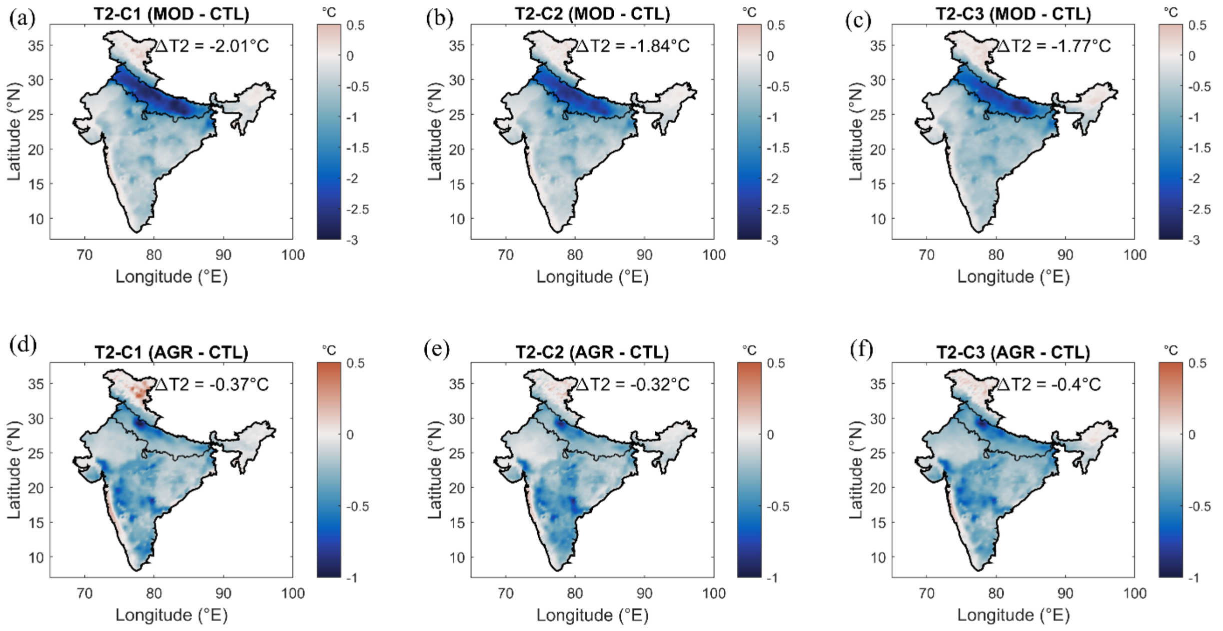

Supplementary Figure 11. Parameterization sensitivity of daily mean temperature response to model-simulated irrigation and census-based irrigation. Difference between daily mean temperature of MOD and CTL experiment during pre-monsoon season (April-May) from 2004-2016 for (a)C1:MYNN3-WDM6-Tiedtke (b)C2:MYNN3-WSM6-KF (c)C3:MYNN3-WSM6-Grell3D. (d-f) Same as a-c but for difference between daily mean temperature of AGR and CTL experiment

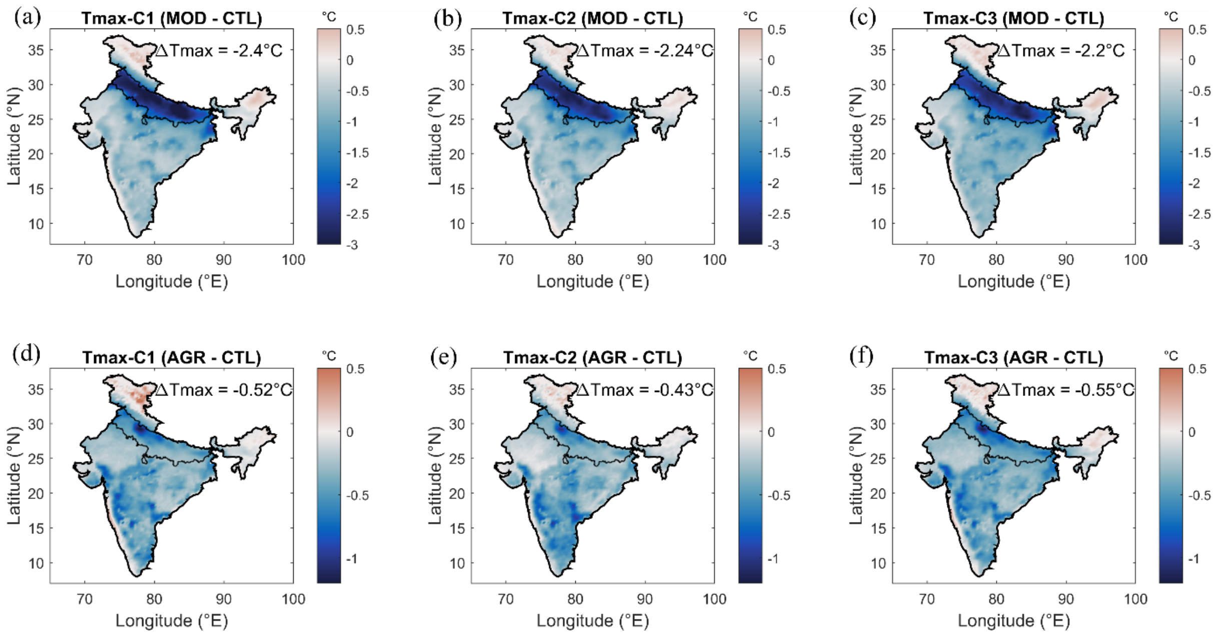

Supplementary Figure 12. Parameterization sensitivity of daily maximum temperature response to model-simulated irrigation and census-based irrigation. Difference between daily maximum temperature of MOD and CTL experiment during pre-monsoon season (April-May) from 2004-2016 for (a)C1:MYNN3-WDM6-Tiedtke (b)C2:MYNN3-WSM6-KF (c)C3:MYNN3-WSM6-Grell3D. (d-f) Same as a-c but for difference between daily maximum temperature of AGR and CTL experiment.

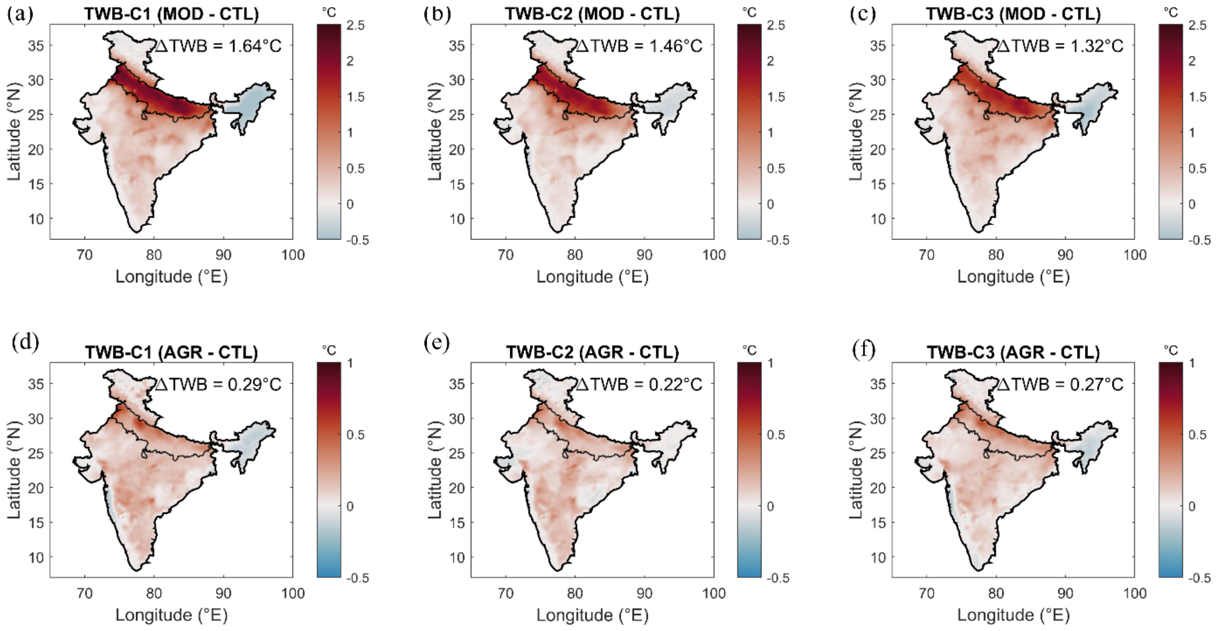

Supplementary Figure 13. Parameterization sensitivity of wet-bulb temperature response to model-simulated irrigation and census-based irrigation. Difference between wet-bulb temperature of MOD and CTL experiment during pre-monsoon season (April-May) from 2004-2016 for (a)C1:MYNN3-WDM6-Tiedtke (b)C2:MYNN3-WSM6-KF (c)C3:MYNN3-WSM6-Grell3D. (d-f) Same as a-c but for difference between wet-bulb temperature of AGR and CTL experiment.

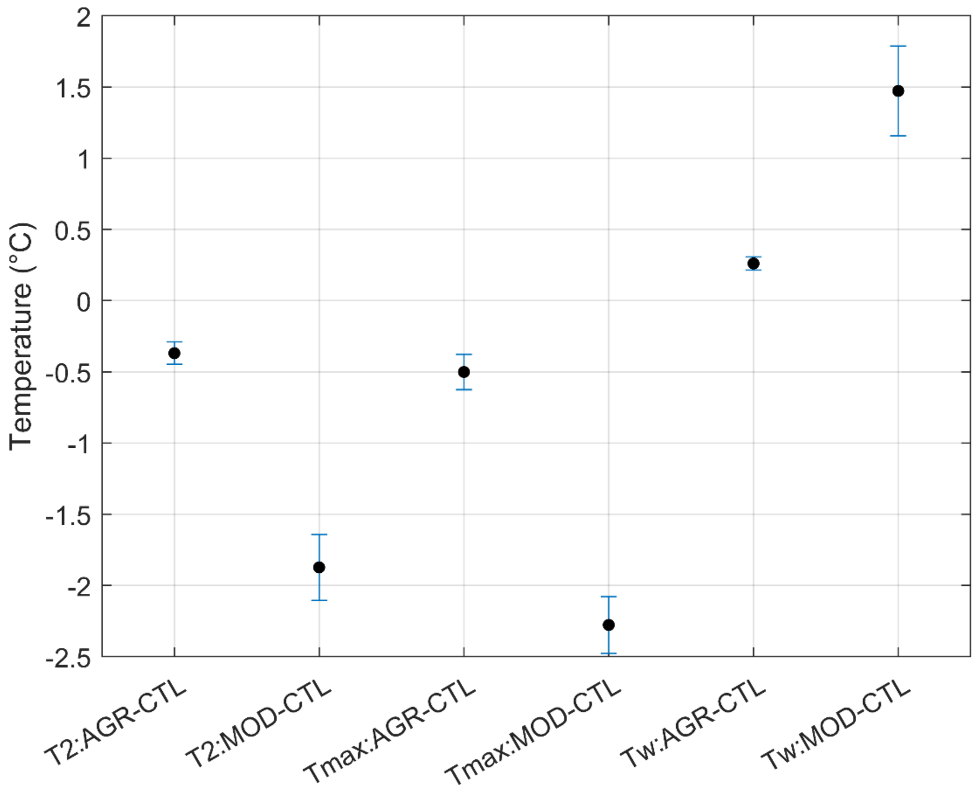

Supplementary Figure 14. Sensitivity to parameterization combination in Indo-Gangetic Plain. Sensitivity of daily mean temperature, daily maximum temperature and wet-bulb temperature spatially averaged over Indo-Gangetic Plain to different parameterization combination for model-simulated (MOD) irrigation and agricultural census-based (AGR) irrigation.

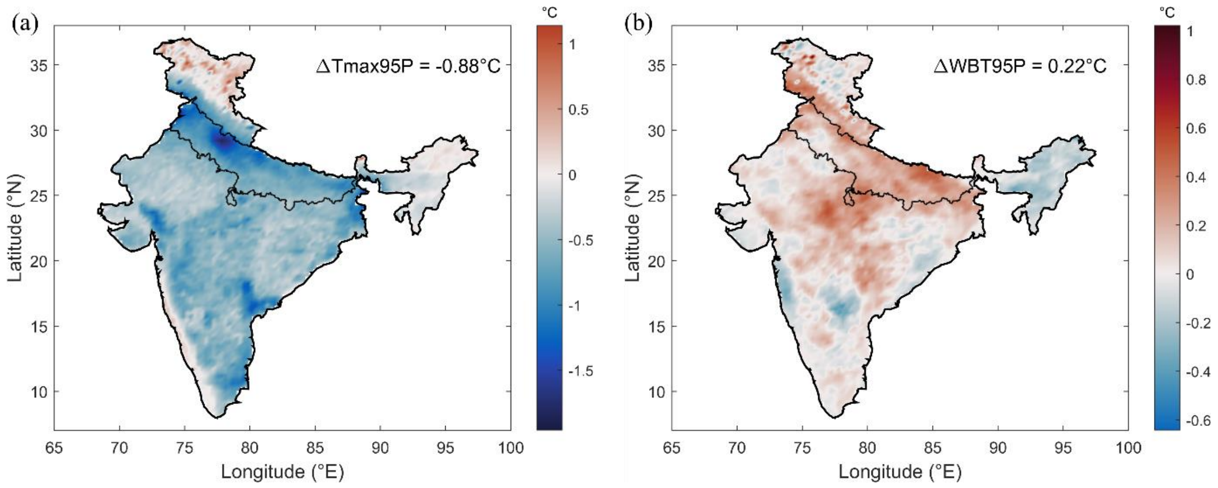

Supplementary Figure 15. Impact of agricultural census based irrigation on 95th Percentiles of daily maximum temperature and wet-bulb temperature. Difference between AGR and CTL experiment during pre-monsoon season (April-May) from 2004-2016 for the 95th percentile of (a) Daily Maximum Temperature (°C) (b) Wet bulb Temperature (°C). CTL represents WRF-CLM4 simulation with no irrigation and AGR represents WRF-CLM4 simulation with agricultural census-based irrigation data.

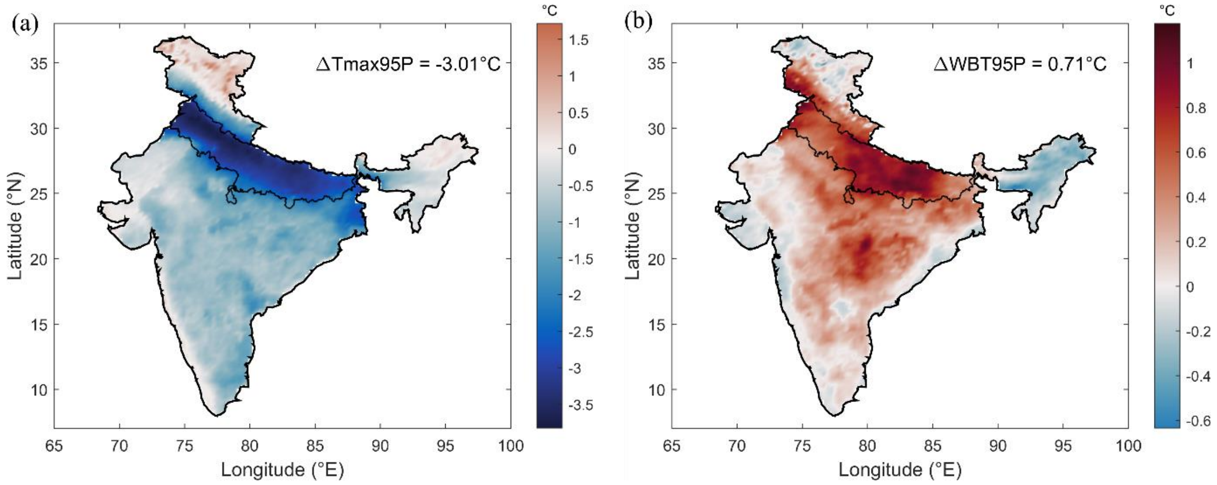

Supplementary Figure 16. Impact of model-estimated irrigation on 95th Percentiles of daily maximum temperature and wet-bulb temperature. Difference between MOD and CTL experiment during pre-monsoon season (April-May) from 2004-2016 for the 95th percentile of (a) Daily Maximum Temperature (°C) (b) Wet bulb Temperature (°C). CTL represents WRF-CLM4 simulation with no irrigation and MOD represents WRF-CLM4 simulation with model-estimated irrigation data.

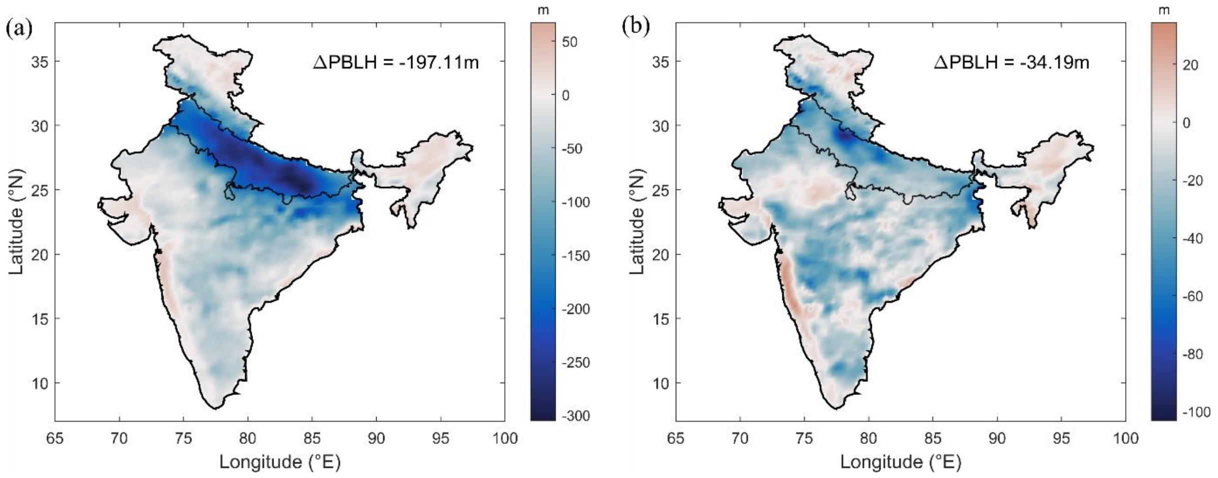

Supplementary Figure 17. Impact of model-estimated irrigation and agricultural census-based irrigation on Planetary Boundary Layer (PBL) height. Difference between (a) MOD and CTL experiment (b) AGR and CTL experiment during pre-monsoon season (April-May) from 2004-2016 for PBL height. CTL represents WRF-CLM4 simulation with no irrigation, AGR represents WRF-CLM4 simulation with agricultural census-based irrigation data and MOD represents WRF-CLM4 simulation with model-estimated irrigation data.

(a)

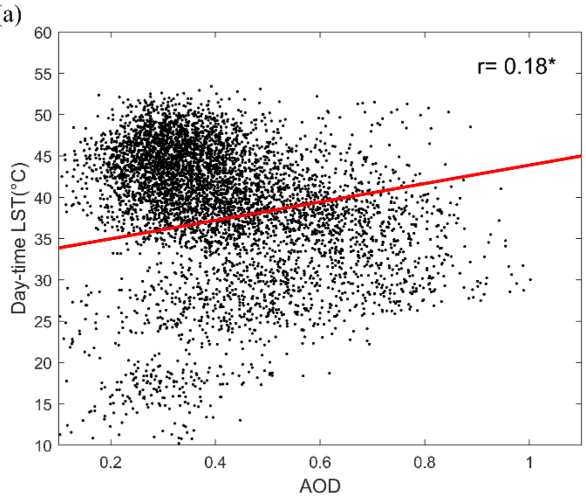

(b)

Granger Causality Test (Punjab & Haryana)	Result (p-value)
LST_Day ~ AOD	AOD Granger-causes LST (0.019)
LST_Day ~ ET	ET Granger-cause LST (0.008)

Supplementary Figure 18. (a) Scatter Plot between Day-time LST and AOD with Pearson's correlation coefficient(r) for India. (b)) Causal relationship between variables for West Indo-Gangetic Plain (Punjab and Haryana): Aerosol Optical Depth (AOD), Evapotranspiration (ET) and day-time land surface temperature (LST) from MODIS using Granger causality test in vector auto-regression model (VAR) framework. The null hypothesis for the test is that lagged Variable1 do not explain the variation in Variable2.

(a)

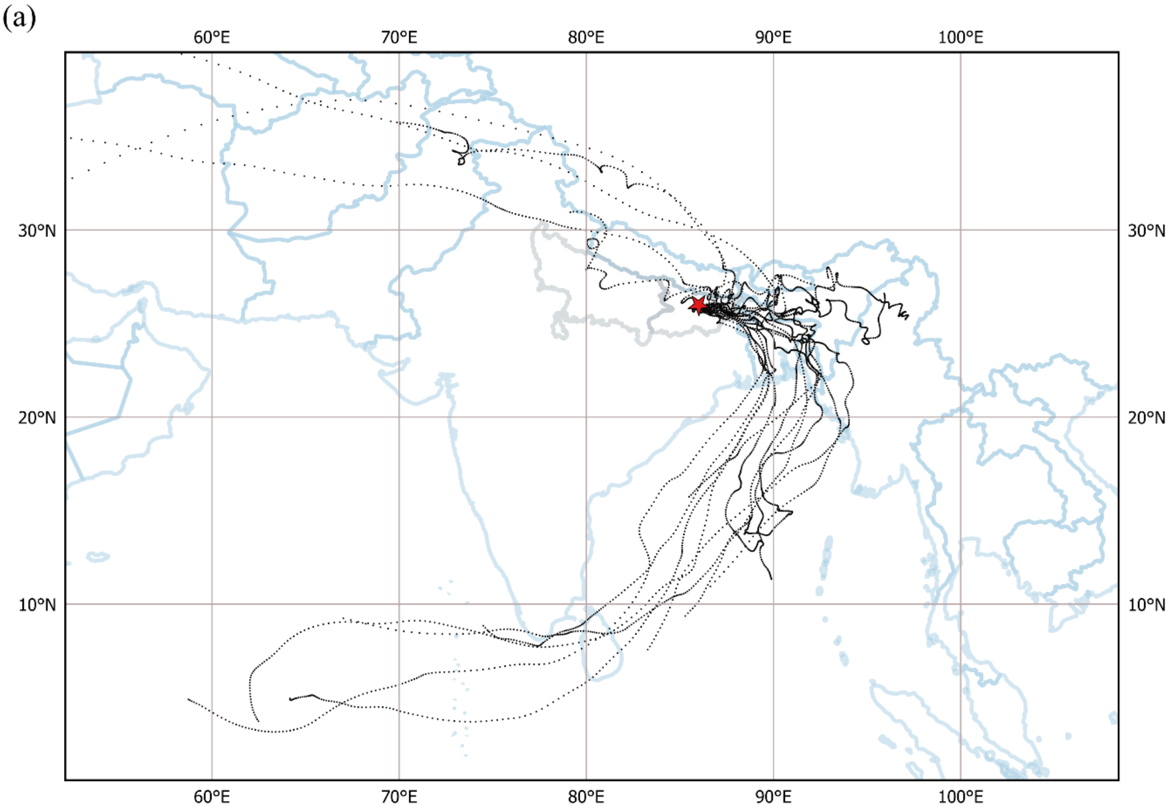

(b)

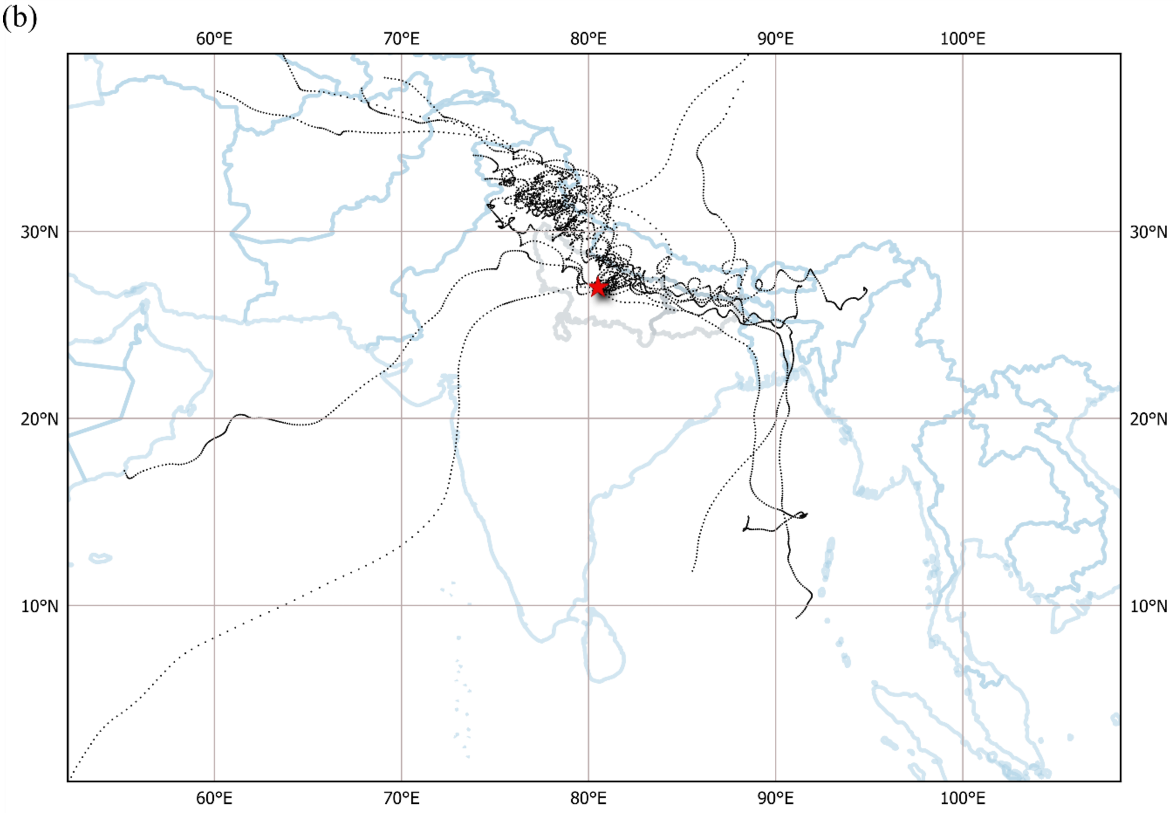

Supplementary Figure 19. Backward air Trajectory Plot using NOAA HYSPLIT Model. Backward trajectory ending at 9:00 UTC (Local time 14:30) for 20 seasonal maximum wet bub temperature day (considering April-May from 2001-2020) with source at (a) Bihar (26°N 86°E) (b) Uttar Pradesh (27.0°N 80.5°E)

Supplementary Tables

Supplementary Table 1. Pre-monsoon season crop calendar for Indo-Gangetic Plain states based on crop production statistics and the annual Indian Agricultural Statistics report . The total water use for each crop is based on Fishman et al. (2015)

**Crops/ States**	**Bihar**	**Haryana**	**Punjab**	**Rajasthan**	**Uttar Pradesh**	**Total wateruse (mm/hectare)**	**Remarks**
**Paddy**	S:Feb-Mar H:Jul-Aug				S:Feb-Mar H:Jul-Aug	**1500**	Pre-monsoon
**Maize**	S:Feb-Mar H:May-Jun	S:Feb-Mar H:May-Jun	S:Feb-Mar H:Apr-May		S:Feb-Mar H:Apr-May	**650**	
**Gram**	S:Feb-Mar H:May-Jun				S:Feb-Mar H:May-Jun	**240**	
**Sunflower**		S:Feb-Mar H:Apr-May	S:Feb-Mar H:Apr-May		S:Feb-Mar H:Apr-May	**500**	
**Sugarcane**	S:Feb-Mar H:Nov-Feb	S:Feb-Mar H:Nov-Feb	S:Feb-Mar H:Nov-Feb	S:Feb-Mar H:Nov-Feb	S:Feb-Mar H:Nov-Feb	**533.33**	1600/3 (seasons=3)

Based on Indian Agricultural Statistics crop calender and pre-monsoon crop production data .

(S=Sowing, H-Harvesting)

Supplementary Table 2. Impact of agricultural census-based irrigation (AGR) on meteorological variables spatially averaged over Indo-Gangetic Plain with significance test for change due to irrigation. Difference between AGR and CTL experiment is taken for pre-monsoon season (April-May) from 2004-2016. Two sample t-test at daily scale and annual scale. The test rejects the null hypothesis at the 5% significance level.

**Indo-Gangetic Plain (All land area)**						
Variable (April-May Annual Mean: 2004-2016)	Irrigation (AGR)	No Irrigation (CTL)	Difference (AGR - CTL)	Percentage Change (%)	P-value (Daily Scale)	P-value (Annual Scale)
T2 (°C)	30.009	30.409	-0.400	-1.314	0.00099	0.34377
Tmax (°C)	36.427	36.975	-0.548	-1.483	0.00033	0.24629
Latent Heat (W/m^2)	35.359	29.718	5.642	18.984	0.00000	0.04339
Sensible Heat (W/m^2)	88.249	90.804	-2.555	-2.814	0.00134	0.18368
PBL Height (m)	1291.748	1325.935	-34.188	-2.578	0.00312	0.26439
Specific Humidity (kg/kg)	0.0091	0.0086	0.0005	5.355	0.00139	0.12680
Relative Humidity (%)	36.083	33.611	2.472	7.355	0.00034	0.16410
Wet-bulb Temp -Tw (°C)	18.319	18.049	0.269	1.493	0.02192	0.26588
Significance Test is based on two sample t-test. The test rejects the null hypothesis at the 5% significance level.						
